# Management of Fatigue in Persons with Multiple Sclerosis

**DOI:** 10.3389/fneur.2014.00177

**Published:** 2014-09-15

**Authors:** Fary Khan, Bhasker Amatya, Mary Galea

**Affiliations:** ^1^Department of Rehabilitation Medicine, Royal Melbourne Hospital, Melbourne, VIC, Australia; ^2^The University of Melbourne, Melbourne, VIC, Australia; ^3^School of Public Health and Preventive Medicine, Monash University, Melbourne, VIC, Australia

**Keywords:** multiple sclerosis, fatigue, disability, rehabilitation outcomes, symptomatic treatment

## Abstract

Fatigue is one of the most common symptoms of multiple sclerosis. Despite advances in pharmacological and non-pharmacological treatment, fatigue continues to be the disabling symptom in persons with MS (pwMS), affecting almost 80% of pwMS. In current practice, both pharmacological and non-pharmacological interventions are used in combination, encompassing a multi-disciplinary approach. The body of research investigating the effect of these interventions is growing. This review systematically evaluated the existing evidence on the effectiveness and safety of different interventions currently applied for the management of fatigue in person with multiple sclerosis in improving patient outcomes, to guide treating clinicians.

## Background

Multiple sclerosis (MS), a chronic progressive demyelinating disease of the central nervous system (CNS), is the commonest cause of chronic neurological disability in young adults (1, 2). It affects approximately 2.5 million persons worldwide and the prevalence of MS in Australia is estimated to be over 20,000 (95.2 per 100,000) persons (2, 3). MS is complex and the exact pathogenesis is unclear. Fatigue is one of the most common symptoms of MS, affecting almost 80% of persons with MS (pwMS) (2), with 55% of pwMS describing it as one of the worst symptoms they experience (1). Fatigue is defined as “*a subjective lack of physical or mental energy that is perceived by the individual or caregiver to interfere with usual and desired activities*” (4). The definitive cause of fatigue in MS is currently unknown, however, it is postulated that MS-related fatigue may result from centrally mediated processes characterized by MS itself, such as demyelination and axonal loss in the CNS or immune actions (*Primary fatigue*) or from MS-related complications (trigeminal neuralgia, spasms, psychological issues, etc.), musculoskeletal problems (pain, posture, gait anomalies, etc.), sleep problems, and medications (*Secondary fatigue*) (5, 6). Experimental studies have shown that fatigue results from reduced voluntary activation of muscles by means of central mechanisms (5). In general, fatigue is a poorly defined construct and hence difficult to measure (7). The MS International Federation recognized two types of fatigue in pwMS, namely: *physical or motor fatigue* (muscle weakness, slurred speech, unable to perform daily tasks, etc.) and *cognitive fatigue* (deterioration of cognitive function such as, reduced reaction time response, alertness during the day, difficulty in thinking, concentration, memory, recall, word finding, etc.) (7, 8). Further, fatigue can be acute (newly occurring in the past 6 weeks) or chronic (lasting longer than 6 weeks) (4). Brañas et al. classifies fatigue experienced by pwMS into: “fatigability” (increased weakness with exercise or as the day progresses) and “lassitude” (abnormal constant and persistent sense of tiredness) (9). In contrast to fatigue in normal people, MS-related fatigue has distinctive characteristics, including: occurs on a daily basis; worse as day progresses; aggravated by heat and humidity; comes on more easily and suddenly; more severe than normal fatigue; and more likely to interfere with role performance and physical functioning (2, 9). Clinically, fatigue may manifest as exhaustion, lack of energy, increased somnolence, or worsening of MS symptoms and activity, and heat typically can exacerbate symptoms (6). The mechanism for fatigue in MS is not known and several different factors are believed to contribute to fatigue (Box 1).

Box 1 **Primary and secondary factors in Multiple Sclerosis fatigue.**

**Primary Factors**

- Immune dysregulation – changes in neuroendocrine function.
- Central nervous system mechanisms – neuronal dysfunction due to immune injury, demyelination and inflammation, impaired innervation, and activation of muscle groups leading to compensatory increase in central motor drive exertion and more energy depletion.
- Endocrine factors – abnormalities in hypothalamic/pituitary/adrenal axis.
- Neurotransmitter dysregulation – dopaminergic, histaminergic, and serotonergic pathways may contribute to fatigue.

**Secondary Factors**

- Physical deconditioning from failure to get adequate exercise.
- Sleep dysfunction – may also be due to nocturnal spasms, pain, incontinence, and depression.
- Pain – sensory disturbances, neuralgia, dysesthesia, and spasms.
- Psychological factors – lack of self-efficacy may increase feelings of fatigue.
- Depression – closely related to poor sleep, pain, and fatigue.
- Medications – can worsen fatigue [antispasticity agents, e.g., Baclofen].

Adapted from MacAllister and Krupp and Kos et al. (5, 10).

Fatigue is prevalent in the MS population and a significant health problem, adversely impacting on activities of daily living, ability to work, social life, and quality of life (QoL) (4). Fatigue has been associated with increased cognitive impairment and on a person’s participatory roles (such as relationships and social integration, etc.) (11). There is strong consensus in literature that many psychosocial factors influence adjustment to fatigue, including the family’s response, coping behaviors, psychological distress, and fatigue-related disability (1, 5). Fatigue is also associated with poorer general health, increased disability, and higher rates of health care utilization (12, 13). In a descriptive study of MS-related disability (*n* = 101), 81% reported fatigue, with those in higher fatigue grades reporting more disability and health care visits, and lower QoL (14). In another study (*n* = 656 patients), 22% reported limitation in level of physical activity, 14% stated it required them to have more frequent rest breaks, and 10% had to discontinue work due to fatigue (15).

Multiple sclerosis can have a fluctuating and often progressive course, making symptomatic management more challenging. The key to symptomatic management of pwMS, including fatigue, is achievement of individualized, patient-centered goals that are set collaboratively with patients, their carers, and the rehabilitation team in a functional context, and should be based on the medical and functional status of each patient (16–18). The quality and quantity of fatigue, and its impact on function is obtained in the patient assessment and history. All contributing factors to fatigue should be identified, and other non-MS causes should be excluded and/or treated appropriately (4). A number of instruments exist in MS literature for the assessment of fatigue and can be subjective (self-reported by patients) and objective (quantified by clinicians through various parameters) (10). Subjective or patient-reported instruments are specifically designed to incorporate a patient’s viewpoint and are more practical for use in clinical settings (10, 19). A list of commonly used subjective measures of MS-related fatigue is provided in Table 1.

**Table 1:** **Commonly used subjective measures of MS-related fatigue**.

Name of scale	Reference	Population	Specified fatigue subscales	No. of items	Scoring
Modified fatigue impact scale	Paralyzed Veterans of America, 1998 (4)	MS	Physical, cognitive, and psychosocial	21	1–7 (Likert scale)
Rochester fatigue diary	Schwid et al. (20)	MS	Lassitude [reduced energy]	12	0–100 (mm) visual analog scale
Fatigue descriptive scale	Iriarte et al. (21)	MS	Spontaneous mention of fatigue, antecedent conditions, frequency, impact on life	5	0–3 (Likert scale)
Fatigue impact scale	Fisk et al. (22)	MS	Physical, cognitive, psychosocial	40	0–4 (Likert scale)
Fatigue assessment instrument	Schwartz et al. (23)	MS, chronic fatigue syndrome, lupus, dysthymia, healthy	Fatigue severity, situation specificity, consequences of fatigue, responds to rest/sleep	29	1–7 (Likert scale)
Single item visual analog scale of fatigue	Krupp et al. (24)	MS, lupus, healthy	Depends on the question	1	0–100 (mm) visual analog scale
Fatigue severity scale	Krupp et al. (24)	MS, lupus, healthy	None	9	1–7 (Likert scale)
Fatigue scale for motor and cognitive functions (FSMC)	Penner et al. (25)	MS	Motor and cognition	20	1–5 (Likert scale)

*Adapted from MacAllister and Krupp (10) and Kos et al. (5)*.

**Figure 1:**
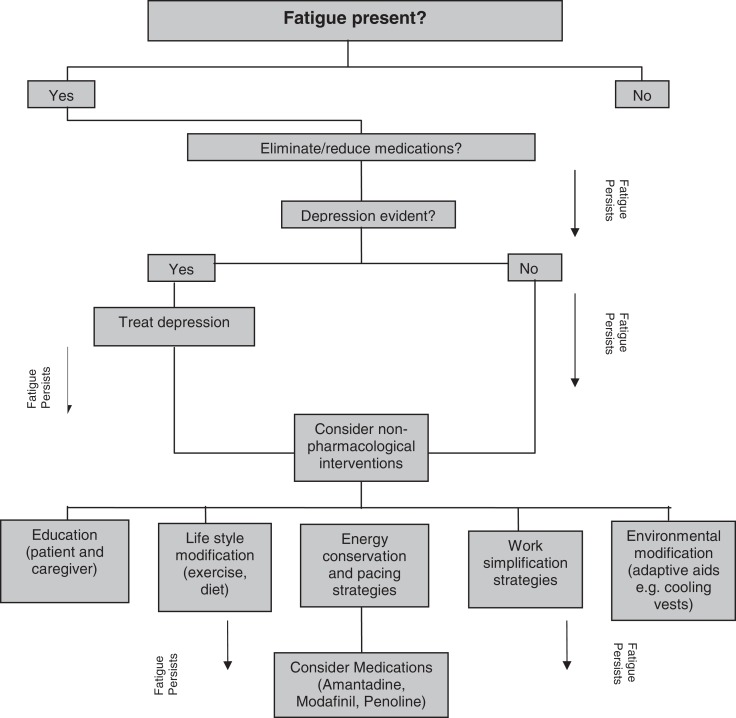
**Clinical decision-making flow chart for treating fatigue in MS**. Adapted from MacAllister and Krupp (10).

The published National Institute for Clinical Excellence (NICE) clinical practice guidelines on the management of MS (26) highlights the significance of diagnosing and treating fatigue as part of the management plan. A clinical decision-making flowchart for managing fatigue in MS (10) is shown in Figure 1. Both pharmacological and non-pharmacological interventions individually or in combination are recommended for the management of fatigue in pwMS. Evidence supporting the efficacy of these interventions in MS-related fatigue is still ambiguous and insufficient (5, 9). The published guidelines acknowledge that the recommendations were mostly driven by the expert opinions rather than by high-quality research-derived evidence (26). Further, interventions for fatigue management in pwMS are still not prescribed in a systematic way (9).

The most commonly used agents for pharmacological treatment for fatigue in pwMS include amantadine, modafinil, and pemoline (9). The NICE guidelines (26) concluded that the efficacy of any pharmacological agents specifically to treat neurological fatigue is yet to be established. Many argue that non-pharmacologic approaches used in isolation and/or in combination with pharmacological agents are the mainstay in the management of fatigue in pwMS (9, 10). Non-pharmacological interventions may include education (e.g., avoid heat, use air conditioners, and cooling gel vests); address lifestyle factors (e.g., diet and exercise; avoid physical activity at midafternoon); pacing (regular rest breaks between activities); energy conservation and work simplification strategies (e.g., use of assistive devices, adaptive equipment, gait aids), and improve aerobic capacity and endurance (e.g., structured exercise programs).

Despite advances in pharmacological and non-pharmacological treatment, MS-related fatigue continues to be the common disabling symptom in pwMS. In current practice, both pharmacological and non-pharmacological interventions are used in combination, encompassing a multi-disciplinary approach. The body of research investigating the effect of these interventions on management of fatigue in MS is growing. The benefit and harms associated with most of these interventions in pwMS needs to be established comprehensively to guide treating clinicians. Therefore, the aim of this review is to systematically evaluate the existing evidence to investigate the effectiveness and safety of interventions for the management of fatigue in pwMS in improving patient outcomes.

## Methods

An integrated approach was used, which included a comprehensive review of literature (peer review and gray literature) documenting interventions currently used in management of fatigue in MS. A comprehensive search of the literature published was undertaken till 6th June 2014 using Medline, Embase, PubMed, and Cochrane Library databases. The search strategy included interventional studies investigating management of fatigue in pwMS, using combinations of multiple search terms for three themes: MS, interventions (pharmacological and non-pharmacological), and fatigue. Medical subject heading (MeSH) search terms were used for all databases and a keyword search was used if the MeSH term was not available. The bibliographies of identified articles were scrutinized for additional references and a manual search of relevant journals was undertaken. A gray literature search using different internet search engines and websites such as: system for Information on Gray Literature in Europe; New York Academy of Medicine Gray Literature Collection, and Google Scholar, was also undertaken. Additional searches of the websites of prominent national and international organizations associated with MS management were conducted to identify relevant reports, health technology assessments, or other related materials.

### Inclusion criteria

Studies that compared various interventions in management of fatigue in pwMS with routinely available local services or lower levels of intervention or placebo, or studies that compared such interventions in different settings or at different levels of intensity, were included. All systematic reviews, meta-analyses, randomized clinical trials (RCTs), and controlled clinical trials (CCTs), quasi-randomized and quasi-experimental designs with comparative controls, and controlled before-and-after studies were included. Whenever RCTs/CCTs were lacking, a search for relevant observational studies was conducted. Studies involving other medical conditions, where data were specifically provided for MS-related fatigue, were also included. Descriptive studies and narrative reviews were explored to identify policies, protocols, and gaps in service provision. Where high-quality systematic reviews or meta-analyses were identified, articles published prior to the date of that review’s search strategy were excluded.

### Exclusion criteria

Limits placed included English-language publication and inclusion of adults aged 18 years and above. Theses, narrative reviews, editorials, case reports, economic evaluation, conference proceedings, and studies evaluating surgical intervention or diagnostic procedures for MS-related fatigue were excluded.

### Study selection

Two authors (Bhasker Amatya and Mary Galea) independently screened and shortlisted all abstracts and titles of studies identified by the search strategy for inclusion and appropriateness based on the selection criteria. Each study was evaluated independently by authors. If necessary, the full text of the article was obtained for further assessment to determine whether the article met the inclusion/exclusion criteria. If no consensus was reached regarding the possible inclusion/exclusion of any individual study, a final consensus decision was made by the third author (Fary Khan). Further information about the complete description of the interventions from the trialists was obtained, where necessary.

### Data extraction

Data extraction was conducted by two authors independently, using a standard *pro forma*. The information obtained from all included studies was: publication date and country, study location, study design, intervention, outcome measures used, and fatigue-related outcomes. Any discrepancies were resolved by all authors re-reviewing the study.

Evidence for all included studies was categorized according to study design using a hierarchy of evidence in descending order and priority were given to the most recently published high-quality systematic reviews or meta-analysis and RCT. Formal levels of evidence were assigned using a standard format defined by National Health and Medical Research Council (NHMRC) pilot program 2005–2006 for intervention studies (Table 2) (27).

**Table 2:** **Designations of “levels of evidence” according to type of research question (27) (intervention studies only)**.^a^

Level	Intervention
I	A systematic review of level II studies
II	A randomized controlled trial
III-1	A pseudo-randomized controlled trial (i.e., alternate allocation or some other method)
III-2	A comparative study with concurrent controls Non-randomized experimental trial Cohort study Case–control study Interrupted time-series with a control group
III-3	A comparative study without concurrent controls Historical control study Two or more single arm study Interrupted time-series without a parallel control group
IV	Case series with either post-test or pre-test/post-test outcomes

*^a^Note that our selection criteria exclude studies at level III-3 and IV*.

## Results

**Figure 2:**
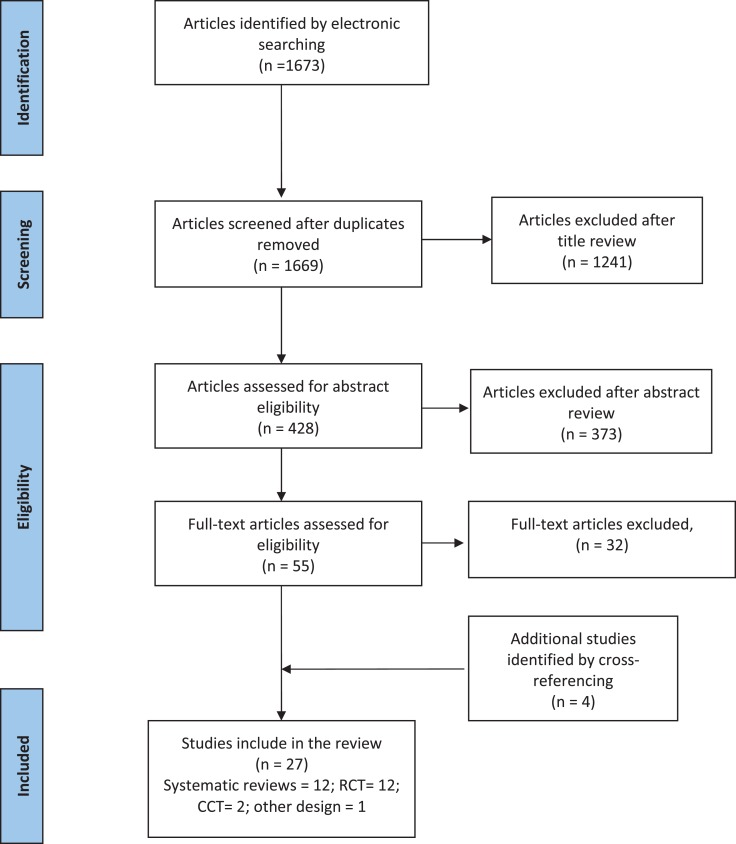
**PRISMA flow diagram showing selection of article review**.

The electronic database search retrieved 1673 published articles on fatigue in MS; 428 articles met title inclusion criteria of which 55 articles met the abstract inclusion criteria and went on to full-text review. Four articles that met the abstract inclusion criteria were identified from the bibliographies of relevant articles. Overall, 27 studies (12 systematic reviews/meta-analyses, 12 RCTs, 2 CCT, and 1 comparative studies) fulfilled the inclusion criteria for this review. The study selection process is summarized in the PRISMA flow diagram shown in Figure 2.

### Evidence for pharmacological interventions for fatigue in persons with MS

Currently, different pharmacological agents are used for treatment for fatigue in pwMS, which include amantadine, modafinil, and pemoline (9, 11). Modafinil, a “wake promoting” agent that selectively works in the hypothalamic pathways used in narcolepsy, has been reported to improve fatigue in progressive MS (5, 9). The efficacy of pemoline, a CNS stimulant, is still unclear (9, 28, 29). Amino pyridines (potassium channel blockers) and amantadine (*N*-methyl d-aspartate receptor antagonist) have been trialed; however, systematic reviews failed to find evidence for efficacy or safety for their use (30). There is empirical support for use of antidepressants in MS-related fatigue, as depression is considered to be one of the major contributing factors (31, 32).

A recently published comprehensive meta-analysis of different interventions (pharmacological and non-pharmacological) included seven RCTs evaluating different medications used for the management of fatigue in pwMS. The authors found weak and inconclusive beneficial effects of pharmacological intervention for MS-related fatigue, with small and non-significant pooled effect sizes (ESs) with a relatively narrow 95% CI (ES = 0.07, 95% CI: −0.22 to −0.37, *p* = 0.63) (11). The pharmacological agents in this review were restricted to Amantadine and Modafinil. Similar inconclusive and insufficient research-derived evidence to support the various pharmacological treatments was reported in another comprehensive systematic review of pharmacological interventions for MS-fatigue published previously (9). The authors systematically reviewed studies investigating only two pharmacological agents: amantadine and pemoline. The studies evaluating the effectiveness of amantadine (four RCTs) showed a pattern in favor of amantadine compared with placebo; however, there was considerable uncertainty about the validity and clinical significance of this finding. Studies investigating efficacy of pemoline (*n* = two RCTs) demonstrated no overall tendency in favor of pemoline over placebo (9). In addition, an excess of reports of adverse effects was noted for pemoline.

One comprehensive systematic review exploring efficacy of different pharmacological treatments on non-specific fatigue in palliative care included 10 studies investigating amantadine (*n* = 6), pemoline, and modafinil in pwMS (33). The authors reported mixed results with weak and inconclusive data. Amantadine (total *n* = 6) was found to demonstrate some improvement in fatigue in pwMS (meta-analysis of three-studies; standard mean difference compared to placebo 1.68). Both pemoline (*n* = 3) and modafinil (*n* = 2) failed to demonstrate a significant effect for management of fatigue in pwMS (33).

Commonly used pharmacological agents for fatigue and MS are summarized in Table 3, along with indications, doses, and side effects.

**Table 3:** **Commonly used pharmacologic treatments for MS-related fatigue**.

Drug	Brand name	FDA indications	Dosage	Common side effects
Amantadine	Symmetrel^®^	Influenza; Parkinson’s Disease	100 mg BID	Livedo reticularis Orthostatic hypotension Peripheral edema Headache Dizziness Nausea Insomnia
Modafinil	Provigil^®^	Narcolepsy; shift-work sleep disorder; excessive daytime sleepiness from OSA not relieved by CPAP	Start 200 mg every morning or at start of shift, may escalate to 400 mg	Anxiety Headache Dizziness Nausea Hypertension Palpitations Insomnia
Armodafinil	Nuvigil^®^	See Modafinil	Start at 150 mg every morning or at start of shift, may escalate to 250 mg	See Modafinil
Pemoline	Cylert^®^	Attention deficit hyperactivity disorder (ADHD)	Starting at 37.5 mg/day and gradually increased by 18.75 mg at 1 week intervals. The maximum recommended daily dose is 112.5 mg	Hepatic dysfunction Insomnia Convulsive seizures Hallucinations Dyskinetic movements of the tongue, lips, face and extremities Abnormal oculomotor function Dizziness Increased irritability; headache; and drowsiness Anorexia and weight loss Nausea and stomach ache

*Adapted from Braley and Chervin (6) and Branas et al. (9)*.

#### Summary

Different pharmacological agents used for treatment of fatigue in pwMS include Amantadine, Modafinil, and Pemoline. There is however, insufficient research-derived evidence to support these pharmacological agents for management of MS related fatigue.

### Evidence for non-pharmacological interventions for fatigue in persons with MS

There is widespread agreement in the literature that, due to the complex, multidimensional, and highly subjective nature of MS-related fatigue, comprehensive goal orientated management programs that incorporate multi-disciplinary (MD) expertise are required, and patients need to be evaluated regularly through appropriate clinical outcome measures (17, 18). The characteristics of the all included studies evaluating non-pharmacological interventions for fatigue in pwMS are summarized in Table 4.

**Table 4:** **Non-pharmacological interventions for fatigue in MS**.

Study, year country	Study design	Potential intervention	Outcome measures for fatigue	Main findings	Level of evidence^a^
**MULTI-DISCIPLINARY (MD) REHABILITATION**					
Khan et al. 2011 (17, 31), Australia	Systematic review, *n* = 10 trials (nine RCTs and one CCT)	Extended MD outpatient rehabilitation	Fatigue, frequency, FIS; MS-related symptom checklist composite score	Fatigue symptoms significantly Improved social functioning and depression	I
		Inpatient MD rehabilitation	MSIS29, VAS	No significant benefits on perceived fatigue or disability level	
**PHYSICAL MODALITIES**					
**Exercise**					
Asano and Finlayson 2014 (11), Canada	Meta-analysis, *n* = 10 RCTs	Various types of exercises (progressive resistance, aerobic, inspiratory exercises, aquatic exercises, vestibular rehabilitation, and leisure exercises)	FSS, MFIS, FIS	Significant beneficial effect in managing fatigue [pooled effect size (ES) was 0.57; 95% CI: 0.10–1.04, *p* = 0.02] ES for the exercise interventions range: −0.24 (95% CI: −1.15 to 0.64) to 2.05 (95% CI: 1.00–3.11)	I
Latimer-Cheung et al. 2013 (42), Canada	Systematic review, *n* = 54 trials (30 evaluating fatigue outcomes: 15 RCTs and 15 other design)	Aerobic fitness; muscle strength (resistance training) and combined	FSS, FIS, MFIS, SF-36 (vitality subscale), PMS (energy and fatigue subscales), MSQL-54 (energy subscale)	Aerobic exercise: significant improvements in some general fatigue symptoms but not specific symptoms after 2–6 months of light to moderate cycling for 40–60 min three times/week; decreases in general, physical, and psychological fatigue symptoms after 8 weeks of moderate-intensity aerobic activities two times/week Traditional resistance training: improvements in general symptomatic fatigue after a 12-week, two times/week resistance training program (8–15 RM); decreased fatigue overall or specifically physical and psychological fatigue after 8 weeks of moderate-intensity resistance training two times/week (6–15 RM) Combined training programs: significant increase in vitality or decrease in fatigue severity after 5–8 weeks of supervised aerobic and resistance training performed at moderate to high intensity; significant improvements in fatigue symptoms or severity after 8–10 weeks of two to three times/week combined training Other types of exercise (sport, yoga, body weight support treadmill training, aquatic exercise, cycling, and Pilates): a significant decrease on at least one indicator of fatigue (general or specific) symptoms	III-1
Andreasen et al. 2011 (39), Denmark	Systematic review, *n* = 21 trials (11 RCTs, 1 CCT, 9 other design)	Endurance training, resistance, training, combined training, or “other” training modalities	FSS, MFI, MFIS, FCMC	Exercise therapy on MS fatigue show heterogeneous results and only few studies have evaluated MS fatigue as the primary outcome All type of exercise modalities have potential to reduce MS fatigue Not clear whether any exercise modalities are superior to others	III-1
Neill et al. 2006 (43), Australia	Systematic review, *n* = 11 trials [combined for MS, rheumatoid arthritis (RA) and systemic lupus erythematosus (SLE); various study design]	Aerobic exercise, resistance training	FIS, FSS, SF-36, POMS, VAS,	Aerobic exercise (home-based or supervised classes) is effective in managing fatigue for some people with MS, RA and SLE Six studies reported statistically significant reductions in fatigue from aerobic exercise interventions Low-impact aerobics, walking, cycling, and jogging were effective interventions	III-1
**Aquatic therapy**					
Kargarfard et al. 2012 (50), Iran	RCT, *n* = 32 women with MS	Aquatic exercise: joint mobility, flexor and extensor muscle strength, balance movements (60 min session three times/week), control group: usual care	MFIS, MSQL-54	Patients in the aquatic exercise group showed significant improvements in fatigue and QoL after 4 and 8 weeks (*p* = 0.002 and <0.001, respectively)	II
Castro-Sánchez et al. 2012 (48), Spain	RCT, *n* = 73 pwMS	Treatment group: aquatic Tai-Chi (40 sessions) (*n* = 36); control group: relaxation (*n* = 37)	FSS, MFIS	Treatment group showed a significant score reduction in fatigue at week 20 (*p* < 0.032) that was maintained at week 24 (*p* < 0.038) An improvement was shown by 48% of the treatment group Significant improvement in pain, spasms, disability, fatigue, and depression was also reported in treatment group	II
Bayraktar et al. 2013 (53), Turkey	CCT, *n* = 23 pwMS	Treatment group: aquatic Tai-Chi (*n* = 15); control group: exercise at home (*n* = 8)	FSS	Significant in reduction in fatigue in the treatment group (*p* < 0.05) Improvement in balance, functional mobility, upper and lower extremity muscle strength was also noted in treatment group (*p* < 0.05)	III-1
**Tai chi**
Castro-Sánchez et al. 2012 (48), Spain	RCT, *n* = 73 pwMS	Treatment group: aquatic Tai-Chi (40 sessions) (*n* = 36); control group: relaxation (*n* = 37)	FSS, MFIS	See “Aquatic Therapy” section above	II
Bayraktar et al. 2013 (53), Turkey	CCT, *n* = 23 pwMS	Treatment group: aquatic Tai-Chi (*n* = 15); control group: exercise at home (*n* = 8)	FSS	See “Aquatic Therapy” section above	III-1
Mills et al. 2000 (56), UK	Comparative study, *n* = 8 pwMS	Tai Chi/QiGong along with the teaching QiGong self-massage. TuiNa and daily home practice for 30 min	POMS, 21-Item symptom checklist	Significant improvements in fatigue post intervention	III-2
**Cooling devices**					
Beenakker et al. 2001 (57), Netherlands	RCT, *n* = 10	Wearing cooling garment for 60 min at 7°C (active cooling); control group: 26°C (sham cooling).	MFIS	Beneficial effect of cooling therapy in reducing fatigue, improving postural stability and muscle strength in pwMS	II
White et al. 2000 (58), USA	RCT, *n* = 6 pwMS	Immersing participants’ lower body regions in water baths at 16–17°C for 30 min before training	FIS	Reduced fatigability during training sessions (*p* < 0.05) Fewer heat-induced symptoms such as ataxia, blurred vision, and foot drop during exercise preceded by cooling	II
**Pulsed electro-magnetic devices**					
Lappin et al. 2003 (60), USA	RCT, *n* = 117 pwMS	“Enermed” – active low-level, pulsed electro-magnetic field device worn up to 24 h daily on one or more acupressure points for up to 4–8 weeks	MSQLI	Statistically significant decreases in fatigue for the intervention groups (0.05) Overall QoL significantly greater on the active device group No treatment effects for bladder control and a disability composite, and mixed results for spasticity	II
Richards et al. 1997 (61), USA	RCT, *n* = 33 pwMS	“Enermed” – see above	Patient-reported performance scales	Significant improvement in the performance scale (PS) combined rating for bladder control, cognitive function, fatigue level, mobility, spasticity, and vision (active group –3.83 ± 1.08, *p* < 0.005; placebo group –0.17 ± 1.07, change in PS scale)	II
**BEHAVIORAL AND EDUCATIONAL INTERVENTIONS**					
Asano and Finlayson 2014 (11), Canada	Meta-analysis, *n* = 8 RCTs	Various types of psychologi-cal/educational interventions (fatigue management program, energy conservation course, CBT, mindfulness intervention)	FSS, MFIS, FIS	Significant beneficial effect in managing fatigue [pooled effect size (ES) was 0.54; 95% CI: 0.30–0.77, *p* < 0.001] ES for the educational interventions range: from -0.16 (95% CI: -0.72 to 0.38) to 1.11 (95% CI: 0.43 to 1.78)	I
Neill J et al. 2006 (43), Australia	Systematic review, *n* = 15 trials (combined for MS, RA and SLE; various study design design)	Education programs, energy conservation, self-management, fatigue management program, CBT	FIS, FSS, SF-36, POMS, VAS,	Behavioral interventions appeared effective in reducing fatigue Education alone or with exercise reduced fatigue and increased vitality in pwMS Rehabilitation program and counseling were effective in reducing fatigue	III-2
**Fatigue management programs**					
Thomas et al. 2013 (70), UK	RCT, *n* = 164 pwMS	Group-based interactive program for managing MS-fatigue [fatigue: applying cognitive behavioral and energy effectiveness techniques to lifestyle (FACETS] (90-min sessions weekly for 6 weeks facili-tated by two health pro-fessionals (*n* = 84); control group (*n* = 80) usual care)	FAI, MSFS	At 1-month post intervention: significant differences favoring the intervention group on fatigue self-efficacy (mean difference = 9; 95% CI 4–14; ES = 0.54, *p* = 0.001). At 4 months follow-up: positive effects of the program still remained significant with moderated effect size (ES = 0.36; *p* = 0.05; mean difference = 6; 95% CI 0–12); significant improvement in fatigue severity was also found in intervention group (*p* = 0.01)	II
Thomas et al. 2014 (64), UK	RCT, *n* = 164 pwMS	Same as above	Same as above	At 1-year follow-up: benefits of the FACETS program for fatigue severity and self-efficacy mostly sustained (ES = -0.29, *p* = 0.06 and 0.34, *p* = 0.09, respectively); additional significant improvements in QoL (*p* = 0.046)	II
Kos et al. 2007 (34), Belgium	RCT, *n* = 51 pwMS	Multi-disciplinary fatigue management program: interactive educational sessions about possible strategies to manage fatigue and reduced energy levels (four 2 h sessions/week) (*n* = 28); control group: placebo	MFIS	No efficacy in reducing the impact of fatigue compared to a placebo intervention program (ES = −0.16)	II
**Energy conservation interventions**					
Blikman et al. 2013 (65), Netherlands	Systematic review, *n* = 6 trials (four RCTs and two CCTs)	Energy conservation interventions: education about balancing, modifying and prioritizing activities, rest, self-care, effective communication, biomechanics, ergonomics, and environmental modification	FIS	Energy conservation interventions were more effective than no treatment in improving subscale scores of FIS: cognitive mean difference (MD = −2.91; 95% CI, −4.32 to −1.50), physical (MD = −2.99; 95% CI, −4.47 to −1.52), and psychosocial (MD = −6.05; 95% CI, −8.72 to −3.37) QoL scores on physical, social function and mental health (also improved significantly in treatment group None of the studies reported long-term results	I
**Mindfulness-based interventions**					
Simpson et al. 2014 (66), UK	Systematic review, *n* = 3 trials (two RCTs and one CCT)	Mindfulness-based interventions: mindful breath awareness, mindful movement, and body awareness or “scanning”	MFIS, POM	Significantly beneficial effect on fatigue scores One RCT found significant post-intervention reduction in fatigue in both overall population and in subgroup analyses of those with pre-intervention impairment (*p* < 0.001 for both). Beneficial effect maintained at 6 months	I
**Cognitive and psychological interventions**					
Moss-Morris et al. 2012 (68), UK	RCT*n* = 40 pwMS	Intervention group (*n* = 23): internet-based cognitive behavior therapy (CBT) – “MS Invigor8” (eight tailored, interactive sessions with a clinical psychologist over 8–10 weeks)Control group (*n* = 17): standard care	MFIS	Significant greater improvements in fatigue severity and impact; and also in anxiety, depression and quality-adjusted life years in treatment group	II
van Kessel et al. 2008 (69), New Zealand	RCT*n* = 72	Treatment group (*n* = 35): CBT (eight weekly sessions)Control group (*n* = 37): relaxation therapy	CFS, MFIS	Both groups showed clinically significant decreases in fatigue Significantly greater improvements in fatigue in treatment group (*p* < 0.02) compared to relaxation therapy group: ES = 3.03 (95% CI 2.22–3.68) for the CBT group across 8 months compared with the relaxation therapy group (ES 1.83; 95% CI 1.26–2.34)	II

*^a^Levels of evidence’ categorized according to National Health and Medical Research Council (NHMRC) pilot program 2005–2006 for intervention studies (23)*.

*CBT, cognitive behavioral therapy; CCT, clinical controlled trial; CFS, Chalder fatigue scale; ES, effect size; 95% CI, 95% confidence interval; FAI, fatigue assessment instrument; FSMC, fatigue scale for motor and cognitive functions; FSS, fatigue severity scale; FIS, fatigue impact scale; MFIS, modified fatigue impact scale; MSFS, multiple sclerosis-fatigue self-efficacy; MSIS, multiple sclerosis impairment scale; MSIS29, multiple sclerosis impact scale; MSQL-54, multiple sclerosis quality of life-54 MFI, multidimensional fatigue inventory; POMS, profile of mood states; QoL, quality of life; RCT, randomized controlled trial; SF-36, short-form health survey-36, VAS, visual analog scales*.

### Multi-disciplinary rehabilitation (level I evidence)

Existing clinical practice guidelines for MS recommend comprehensive, co-ordinated MD care, including symptomatic management, and appropriate follow up, education, and support for patients and carers (26). MD rehabilitation, a co-ordinated delivery of patient-centered, time-based, functionally oriented intervention/s by two or more disciplines (such as physiotherapy, occupational therapy, social work, psychology, and other allied health, nursing), under medical supervision (17), should be the best approach in symptomatic management in MS, including fatigue (5, 34). A systematic review of MD rehabilitation in MS (17), found a “strong evidence” to support MD rehabilitation in producing short-term gains at the levels of activity (disability) and participation in patients with MS. Of the 10 included trials, fatigue was considered in only two studies evaluating outpatient and home-based rehabilitation programs. A CCT (35) evaluating the influence of an extended MD outpatient rehabilitation found that fatigue symptoms were significantly reduced in the treatment group compared to the control group at 1-year follow-up (*p* = 0.004). Similar result was reported in another RCT evaluating impact of outpatient MD rehabilitation. The authors reported that a 12-week rehabilitation program significantly reduced fatigue and improved social functioning and depression (*p* < 0.001) (36). There was no convincing evidence regarding the effectiveness of inpatient MD rehabilitation programs for management of fatigue (17). An RCT investigating MD inpatient rehabilitation did not find any significant benefits of such a program on disability level or perceived fatigue (37).

### Specific rehabilitation interventions

The cause and effect of fatigue are considered to be multidimensional and its impact extends from general everyday activity to overall QoL of pwMS (11, 38). Improving or restoring physical and psychosocial abilities and education have been proposed to counteract many MS-fatigue-related consequences. A rehabilitation approach to fatigue management in pwMS includes a spectrum of interventions, which have been examined in several published reviews. However, many of these interventions have not yet been included routinely in comprehensive MD rehabilitation programs, and few studies show their implementation. The existing evidence for various specific rehabilitation interventions for fatigue management in pwMS is summarized below.

### Physical therapeutic modalities

Physical therapeutic modalities are considered to be one of the most efficient strategies in rehabilitation of MS patients in improving or restoring physical abilities. However, its role in MS-related fatigue management has been controversial. In past years, pwMS were advised not to participate in physical activities because it was believed to lead to worsening of symptoms or fatigue (15, 39, 40). However, recent studies on exercise therapy in MS have demonstrated that it results in substantial long-term reduction in functional limitations and enhanced QoL, and have the potential to reduce fatigue in pwMS (39).

### Exercise (level I)

Exercise therapy is a core rehabilitative measure, which aims to improve motor functions (such as co-ordination, fine-movements), balance, gait, and reduction of MS-related symptoms. Compared with the other interventions, exercise has been more frequently investigated for MS-related fatigue, which has resulted in several systematic reviews/meta-analyses evaluating various exercise modalities for the management of fatigue (11, 41–45). A wide range of exercise interventions were investigated, which included resistance training, endurance training, aquatic exercises, leisure activities, and a combination of two or more exercise modalities. In a recently published systematic review, Asano and Finlayson reported strong evidence for exercise-based rehabilitation in terms of reducing severity of patient-reported fatigue (11). Although there was heterogeneity among the included trials (*n* = 10 studies; *p* = 0.003), exercise interventions were still found to have a significant beneficial effect in managing fatigue in pwMS (pooled ES was 0.57; 95% CI: 0.10–1.04, *p* = 0.02). The authors stated that the extent of the intervention effects varied considerably and only a certain group of patients (younger, with stable MS) appear to experience benefit. For other MS subgroups, such as older adults or those with progressive MS and/or severe disability, there was no evidence of benefit. Further, it was not possible to identify which types or components or intensity of exercise achieved benefits for fatigue management. Another meta-analysis (*n* = 17 RCTs), demonstrated a similar positive effect of exercise interventions for MS-related fatigue (45). The authors showed that exercise training was associated with a significant reduction in fatigue among pwMS (weighted mean ES = 0.45; 95% CI = 0.22–0.68, *p* ≤ 0.001) (45). A systematic review by Andreasen et al. assessed the beneficial effect of different exercise categories separately; these included endurance training, resistance training, combined, or “other” training modalities (39). The authors, consistent with other reviews, found marked heterogeneity among the trials, as only a few studies evaluated MS fatigue as the primary outcome and many studies included non-fatigued MS patients. Overall, all type of exercise interventions were shown to have the potential to reduce MS fatigue (39). The authors concluded that, compared to other exercise modalities, endurance training was studied more frequently (*n* = 11 studies) and showed more consistent positive effects (39).

Several reviews evaluated exercise therapy for MS in general (42, 44, 46) and reported strong evidence in favor of exercise therapy compared to no exercise therapy, in terms of muscle power, exercise tolerance, and mobility-related activities. Conversely, subgroup analysis of results on fatigue showed mixed results. One study found that neurophysiologically based physiotherapy or a combined training program (physiotherapy plus aerobic training) were associated with significant improvement in impairment and fatigue (47).

#### Summary

Overall, the evidence regarding exercise modalities for MS-related fatigue was inconsistent and data for an optimal type or intensity of exercise intervention are still insufficient. Some types of exercise interventions which include endurance and a resistance-training component may have potential beneficial effects on fatigue reduction in pwMS.

### Aquatic therapy (level II)

Few studies have evaluated aquatic therapy, which aims to reduce resistance of movements and gravity by exercising in water (pool therapy, hydrotherapy, balneotherapy), for management of fatigue in pwMS (48–52). There is evidence from two RCTs showing beneficial effects of an aquatic exercise program for MS-related fatigue. One RCT examined the effectiveness of a supervised 8-week aquatic exercise training program (60 min session, three times a week) on fatigue and health-related QoL in women (*n* = 32) with MS (50). The participants in the aquatic exercise group showed significant improvements in fatigue and QoL after 4 and 8 weeks compared with the control group (50). Another RCT (*n* = 73) suggested that a structured aquatic exercise (Ai Chi) program for 20 weeks (40 sessions) improved fatigue, pain, spasms, disability, and depression in pwMS (48). Bayraktar et al. investigated the effects of a similar aquatic exercise program (Ai Chi) in a CCT (*n* = 23) on balance, functional mobility, strength, and fatigue in ambulatory pwMS (53). The authors reported significant improvements in fatigue, static standing balance, functional mobility, and upper and lower extremity muscle strength in the treatment group (*p* < 0.05) (53).

#### Summary

Aquatic exercise training can improve fatigue and other MS-related symptoms, function and quality of life of pwMS and could be considered for inclusion in management programs.

### Tai chi (level II)

Tai Chi is gaining momentum in rehabilitation settings and can improve balance, posture, muscle strength, psychological issues (stress reduction, and decreased anxiety, depression and mood disturbance) and general well-being in people with various medical conditions (54, 55). The effects of Tai Chi on fatigue in pwMS have been evaluated in only a few studies. Two trials (one RCT and one CCT) (also reported under aquatic exercise) investigated the effectiveness of Tai Chi aquatic exercise program in reducing symptoms, including fatigue and improving physical function in pwMS (48, 53). There was a significant reduction in fatigue in individuals with MS participating in the Tai Chi classes as compared to the control group (see above in section “Aquatic Therapy”). Another comparative study found that practicing Tai Chi for 2 months daily was associated with some improvements in fatigue and significant improvements in balance and depressive symptoms in pwMS (56).

#### Summary

There is limited evidence suggesting the effectiveness of Tai chi in improving fatigue symptoms in pwMS. Further studies with a larger sample size are needed to confirm the potential effectiveness of Tai chi in fatigue management in pwMS.

### Cooling therapy (level II)

Physiological approaches such as cooling techniques using different cooling temperatures and durations have been tested for symptomatic management in heat-sensitive pwMS. Beenakker et al. conducted a RCT showing a beneficial effect of cooling therapy in reducing fatigue, improving postural stability, and muscle strength in pwMS when wearing a cold vest with active cooling (7°C, 60 min) (57). Another study investigating the effects of immersing participants’ lower body regions in water baths at 16–17°C for 30 min before training, found that fatigability significantly reduced in these patients during training sessions (58). These effects of cooling on functional improvements are most probably due to temperature-induced changes (Uhthoff phenomenon) in central motor conduction in demyelinated fibers (59).

#### Summary

Pre-cooling or cooling during and after therapy may decrease fatigue and increase the effect of active physical training in thermo sensitive pwMS. However, the evidence is limited and unclear. Further research is required to identify who will benefit from these techniques.

### Pulsed electro-magnetic devices (level II)

Low-level pulsed electro-magnetic field devices have been investigated in a few trials and have shown positive effects in reducing for MS-related fatigue (60–62). A multi-center RCT (*n* = 117) found that wearing an active low-level, pulsed electro-magnetic field device on one or more acupressure points daily for up to 4–8 weeks, significantly decreased fatigue (60). Similar positive results were reported in another RCT (*n* = 33) conducted earlier using the similar device (61). The clinical effects in these trials were small and long-term follow-up data were lacking.

#### Summary

Exposure to pulsing, weak electromagnetic fields can alleviate fatigue symptoms in pwMS, however, additional research is needed into the feasibility and long-term use of these devices, due to limited access and cost of devices.

### Behavioral and educational interventions

Several published reviews and studies have examined the effectiveness of various types of behavioral and/or educational interventions for management of fatigue in pwMS, which included group fatigue management programs, energy conservation programs, and psychotherapies [e.g., cognitive behavioral therapy (CBT) and mindfulness-based intervention]. A meta-analysis investigated overall effectiveness of different types of educational programs on reducing the impact or severity of self-reported fatigue in pwMS (11). The authors included eight RCTs, involving 662 pwMS. Educational interventions included a fatigue management program, energy conservation programs, mindfulness interventions, and CBT. The authors found significant global improvement with a large pooled treatment ES for the educational interventions of 0.54 (95% CI: 0.30–0.77 *p* < 0.001; range: −0.16 to 1.11) (11).

### Fatigue management programs (level II)

A number of structured fatigue management programs have been explored in pwMS and most appeared effective in reducing fatigue. A multi-centered parallel arm RCT (*n* = 164) evaluated the effectiveness of a group-based program for managing MS-fatigue [fatigue: applying cognitive behavioral and energy effectiveness techniques to lifestyle (FACETS)], which was based upon a conceptual framework integrating elements from cognitive behavioral, social-cognitive, energy effectiveness, self-management, and self-efficacy theories (62). The program consisted of interactive group sessions and activities (90-min sessions weekly for 6 weeks) and was facilitated by two health professionals (such as occupational therapists, nurses, or physiotherapists). The authors found significant differences favoring the intervention group on fatigue self-efficacy at 1 month follow-up (mean difference = 9; 95% CI 4–14) with a large ES (ES = 0.54, *p* = 0.001). At 4 months follow-up, the positive effects of the program still remained significant with a moderate ES (ES = 0.36; *p* = 0.05; mean difference = 6; 95% CI 0–12). In addition, significant improvement in fatigue severity was also found in the intervention group (*p* = 0.01) at 4 months follow-up (62). In a 1-year follow-up study by the same authors, the findings showed that the benefits of the FACETS program for fatigue severity and self-efficacy were mostly sustained, with a slight reduction in standardized ESs (ES = −0.29, *p* = 0.06 and 0.34, *p* = 0.09, respectively) with additional significant improvements in QoL (*p* = 0.046) (63). Another RCT (*n* = 51) evaluating the efficacy of a MD fatigue management program in pwMS, however, showed no efficacy in reducing the impact of fatigue compared to a placebo intervention program (34). The MD fatigue management program comprised interactive educational sessions about possible strategies to manage fatigue and reduced energy levels (2 h sessions weekly for 4 weeks).

#### Summary

A structured fatigue management program based on psychological approaches delivered by health professionals can be effective in reducing fatigue severity and increasing fatigue self-efficacy for pwMS. It can be clinically beneficial and can be readily incorporated into existing services.

### Energy conservation interventions (level I)

A systematic review evaluated the effectiveness of energy conservation treatment for fatigue and QoL in pwMS (64). The authors included six trials (four RCTs and two CCTs) involving 494 participants, which evaluated different energy conservation interventions based on evidence-based protocols, which included education about balancing, modifying and prioritizing activities, rest, self-care, effective communication, biomechanics, ergonomics, and environmental modification. The results were mixed due to heterogeneity among the included studies. Meta-analysis of two high-quality studies showed that energy conservation interventions treatment was significantly more effective than no treatment (waiting controls) in reducing the impact of fatigue and in improving QoL in the short-term. This was further supported by the qualitative best-evidence synthesis of the other studies showing moderate to strong evidence (64). There was no evidence that MD fatigue management programs were more effective than placebo for any fatigue-related outcome.

#### Summary

Energy conservation interventions can be effective in reducing the impact of fatigue and improving QoL in pwMS in the short-term. More high-quality RCTs are still needed to investigate the usefulness of these treatments in the longer-term.

### Mindfulness-based interventions (level I)

Mindfulness-based interventions have become increasingly popular in various areas of chronic disease management such as depression, stroke, chronic pain, etc. (65). Mindfulness-based interventions include a wide range of interventions, such as meditation, relaxation, and breathing techniques, yoga, Tai Chi, hypnosis, visual imagery, and spirituality (55). There are few studies evaluating the effects of the mindfulness-based approach in alleviating fatigue in pwMS. A recently published systematic review of mindfulness-based interventions found only three trials (two RCTs and one CCT) involving 183 participants (65). All trials emphasized on mindful breath awareness, mindful movement, and body awareness or “scanning.” All three studies measured the effect of intervention on fatigue and found a significantly beneficial effect of intervention on fatigue scores. One included RCT found a significant post-intervention reduction in fatigue in both the overall population and in subgroup analyses of those with pre-intervention impairment. This beneficial effect was maintained at 6 months (65).

#### Summary

Mindfulness-based interventions can be beneficial for fatigue management in pwMS and are conceptually appealing. These interventions could be considered in a patient management plan.

### Cognitive and psychological interventions (level II)

Several studies have investigated cognitive training in pwMS aiming mainly to improve attentional deficits, communication, and memory (66). Overall evidence for beneficial effects of psychological interventions in management of fatigue in pwMS is scarce. A systematic review reported that cognitive behavioral approaches were beneficial in the treatment of depression and in helping people adjust to, and cope with having MS (66). However, the authors did not find any studies focusing on psychological approaches to managing fatigue in pwMS. Findings from a few studies evaluating fatigue as a secondary outcome showed inconclusive and/or non-significant improvements in fatigue management (66).

A recent RCT (*n* = 40) showed that an internet-based cognitive behavior therapy (CBT) program – “MS Invigor8” was an effective treatment for MS-related fatigue (67). The CBT included eight tailored, interactive sessions with a clinical psychologist over 8–10 weeks. The treatment group reported significantly greater improvements in fatigue severity and impact as well as in anxiety, depression and quality-adjusted life years (67). Another RCT (*n* = 72) showed significantly greater improvements in fatigue in pwMS after eight weekly sessions of CBT (*p* < 0.02) compared to relaxation therapy (68). However, both groups showed clinically significant decreases in fatigue. ESs for reduction in fatigue from baseline to the end of treatment were 3.03 (95% CI 2.22–3.68) for the CBT group across the 8 months compared with the relaxation therapy group (ES 1.83; 95% CI 1.26–2.34) (68).

#### Summary

Psychological interventions, particularly CBT, can be a clinically and cost-effective treatment for MS fatigue. There has been a growing interest in these interventions as a means of empowering patients, improving symptoms and overall quality of life. Additional studies are warranted, particularly those that include larger numbers of people and longer term follow-up.

## Summary

Fatigue, a multidimensional, complex, and highly subjective symptom, is one of the most frequent symptoms of MS patients. It is associated with several factors or mechanisms. There is a continuing need for a comprehensive, multi-disciplinary long-term management, which includes both pharmacological and non-pharmacological interventions. This systematic review provides an evidence-based overview of the effectiveness of different interventions (pharmacological and non-pharmacological) currently used to alleviate fatigue in pwMS. It highlights the lack of, methodologically robust trials to evaluate effectiveness of MS fatigue management interventions.

Despite many interventions (both pharmacological and non-pharmacological) used for the management of fatigue in pwMS, effects of these vary considerably and any beneficial effect was at best modest and/or is yet to be established. Non-pharmacological interventions (both exercise and psychological/educational interventions) appear to have a stronger and more significant favorable effect on reducing the impact or severity of fatigue compared to commonly prescribed pharmacological agents.

In conclusion, there is increasing awareness of the role of both pharmacological and non-pharmacological interventions in early and long-term management of fatigue in pwMS. Although this review highlights the lack of high-quality studies evaluating fatigue management strategies in pwMS (types, settings, components, modalities, and duration of therapy), it adds to the existing evidence by providing structured pre-defined “level of evidence” to support different interventions for the management of fatigue in this population. The findings from this review suggest that non-pharmacological approaches used in isolation and/or in combination with pharmacological agents should be the mainstay of management of fatigue in pwMS. Further studies across the broad range of interventions for the management of fatigue in MS are warranted, using high-quality research approaches.

## Conflict of Interest Statement

The authors declare that the research was conducted in the absence of any commercial or financial relationships that could be construed as a potential conflict of interest.
